# 3D *in-vitro* cultures of human bone marrow and Wharton’s jelly derived mesenchymal stromal cells show high chondrogenic potential

**DOI:** 10.3389/fbioe.2022.986310

**Published:** 2022-09-26

**Authors:** Erwin Pavel Lamparelli, Maria Camilla Ciardulli, Valentina Giudice, Pasqualina Scala, Rosa Vitolo, Tina Patricia Dale, Carmine Selleri, Nicholas Robert Forsyth, Nicola Maffulli, Giovanna Della Porta

**Affiliations:** ^1^ Department of Medicine, Surgery and Dentistry, University of Salerno, Baronissi, SA, Italy; ^2^ Hematology and Transplant Center, University Hospital “San Giovanni di Dio e Ruggi D’Aragona”, Salerno, SA, Italy; ^3^ Guy Hilton Research Centre, School of Pharmacy and Bioengineering, Keele University, Stoke-on-Trent, Staffordshire, United Kingdom; ^4^ Centre for Sport and Exercise Medicine, Barts and The London School of Medicine, Queen Mary University of London, London, United Kingdom; ^5^ Research Centre for Biomaterials BIONAM, Università di Salerno, Fisciano, SA, Italy

**Keywords:** 3D cell culture, human bone marrow mesenchymal stem cells, human Wharton’s jelly mesenchymal stem cells, hTGF-β1, chondrogenesis, cytokines

## Abstract

In this study, chondrogenic potentials of 3D high-density cultures of Bone Marrow (BM) and Wharton’s Jelly (WJ)-derived mesenchymal stromal cells (MSCs) was investigated by chondrogenesis- and cytokine-related gene expression over a 16-day culture period supplemented with human transforming growth factor (hTGF)-β1 at 10 ng/ml. In BM-MSC 3D models, a marked upregulation of chondrogenesis-related genes, such as *SOX9*, *COL2A1*, and *ACAN* (all *p* < 0.05) and formation of spherical pellets with structured type II collagen fibers were observed. Similarly, WJ-based high-density culture appeared higher in size and more regular in shape, with a significant overexpression of *COL2A1* and *ACAN* (all *p* < 0.05) at day 16. Moreover, a similar upregulation trend was documented for *IL-6* and *IL-10* expression in both BM and WJ 3D systems. In conclusion, MSC-based high-density cultures can be considered a promising *in vitro* model of cartilage regeneration and tissue engineering. Moreover, our data support the use of WJ-MSCs as a valid alternative for chondrogenic commitment of stem cells in regenerative medicine.

## Graphical Abstract

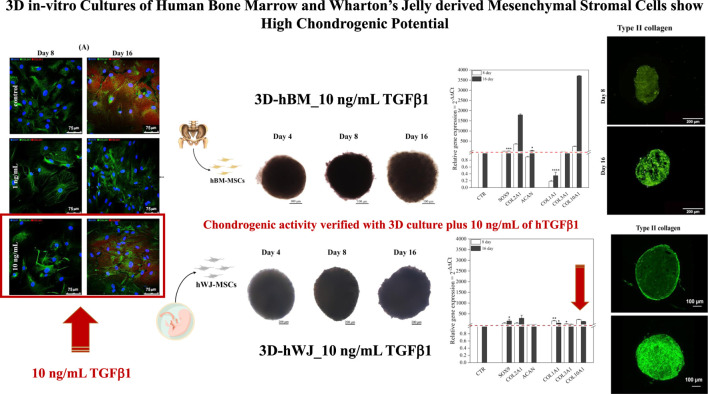

## Introduction

Hyaline cartilage, a specialized connective tissue with the principal task to provide a regular and lubricated surface in joints, helps in preventing the wear process (Sophia Fox et al., 2009) and in minimizing subchondral bone pressure by equally distributing load forces (Bhosale and Richardson, 2008). This tissue is composed of mature cells with poor mitotic potential, termed chondrocytes, and a complex extracellular matrix (ECM) containing type II collagen and aggrecan (ACAN), the main histological cartilage markers (Coburn et al., 2013; Maldonado and Nam, 2013). Type II collagen fibers provide tensile strength, are histologically highlighted with picrosirius red staining (PSR), and are visible under polarized light because of their birefringence properties (Schmitz et al., 2010a). Conversely, fibrous cartilage contains type I collagen and shows different biomechanical behavior (Roberts et al., 2009a). Cartilage lesions represent a substantial challenge for modern orthopedics as they do not spontaneously heal due to the absence of vascularity and innervation, and untreated lesions may have an unfavorable prognosis with the development of osteoarthritis (Grässel and Muschter, 2020).

3D *in vitro* models of chondrogenic differentiation using human stem cells are becoming the most utilized model to study healing and regenerative events. Human mesenchymal stromal cells (MSCs) derived from bone marrow (hBM-MSCs) have a well-described chondrogenic potential (Bernardo et al., 2007). MSCs, multipotent progenitor cells with self-renewal potential, can differentiate in several tissue types, such as muscle, adipose, and trabecular bone tissue; however, MSC harvesting from BM requires invasive procedures and the mesenchymal fraction is low (Mushahary et al., 2018). For these reasons, alternative MSC sources have been identified, such as adipose and umbilical cord tissue comprised of Wharton’s Jelly and umbilical cord blood (Johnstone et al., 1998; Wagner et al., 2005). However, Wharton’s Jelly-derived mesenchymal stromal cell (hWJ-MSCs) chondrogenic potential remains poorly investigated, despite those cells have a superior proliferative capacity, making them extremely valuable in tissue engineering context (Ciardulli et al., 2020a). Moreover, MSCs could modulate microenvironment composition as described in myelodysplasia, where they can contribute to hematopoietic stem cell growth inhibition by secretion of pro-inflammatory cytokines, especially transforming growth factor-beta (TGF-β) (Patel et al., 2021). Other cytokines and growth factors are important in modulating chondrogenesis in health and disease, as pro-inflammatory cytokines, such as interleukin (IL)-1β, that induces chondrocyte-mediated ECM proteolysis, or IL-6, can significantly reduce proliferation potential of chondrocytes and favor osteoarthritis events (Zhou et al., 2018; Razmara et al., 2019; Jafri et al., 2020a; Bhogoju et al., 2022). Conversely, anti-inflammatory cytokines, such as IL-4 and IL-10, are upregulated by BM-MSC models, induce physiological cartilage turnover over osteoarthritis events, and reduce inflammatory processes of the synovia (Jafri et al., 2020b).

Interestingly, TGF-β, both hTGF-β1 and hTGF-β3, is the principal growth factor used for establishing chondrogenesis *in vitro* (Danišovič et al., 2012a). In particular, hTGF-β1 is the most investigated, both *in vitro* and *in vivo* experiments (Huang et al., 2004a) (Roberts et al., 2009b); however, hTGF-β1 has a short half-life when supplemented in culture medium that might be overcome using microencapsulation with biopolymeric vehicles (Wakefield et al., 1990; Palazzo et al., 2021). hTGF-β1 promotes chondrogenesis through activation of different intracellular signaling pathways, including mitogen activated protein (MAP) kinases, p38, or extracellular signal-regulated kinase-1 (ERK-1) (Tuli et al., 2003). Conventional monolayer cultures (2D) are considered unsuitable for MSC differentiation towards chondrogenic phenotype and chondrocyte expansion; furthermore, 2D culture is often reported to promote dedifferentiation processes of chondrocytes and acquisition of fibroblast phenotype (Caron et al., 2012a).

Recently, stem cell 3D cultures have been proposed for chondrogenesis studies because they better mimic cartilage microenvironment where chondrocytes reside. The two predominant strategies are either utilization of a biomaterial scaffold to support seeding and subsequent differentiation, or alternatively, scaffold-free techniques, such as 3D high-density aggregate culture with several advantages (Costa et al., 2016). Suitable biomaterials composed by natural or ECM analog components can enhance cell functions, support differentiation toward specific phenotypes, and modulate immune responses (Nii and Katayama, 2021). In the context of cartilage regeneration, synthesis of advanced biomaterials with strict biomechanical properties, such as alginate hydrogels, is important to develop new surgical adjuvants for *in vivo* implantation (Bidarra et al., 2014; Cao et al., 2014; Baldino et al., 2021); however, clinical application is limited because of potential calcification processes (Ma et al., 2005). Chitosan, another polysaccharide, seems less suitable as a scaffold material as chondrocytes display reduced proliferation abilities likely due to its cationic properties (Sechriest et al., 2000). Conversely, collagen hydrogels can be an appropriate support for drug delivery system and/or stem cells local implantation (Lamparelli et al., 2021; 2022b), whereas, collagen-hyaluronic acid derivatives may be proposed as implantable 3D scaffold (Lamparelli et al., 2022a).

Polylactic-co-glycolic acid (PLGA), a synthetic biocompatible material, is rapidly degraded and releases acidic components that can cause inflammatory responses. Moreover, PLGA has no natural cell recognition sites, giving poor cell affinity (Mao et al., 2021). Conversely, poly (l-lactic acid) PLA is more effective in preventing *in vivo* hypertrophic drift of BM-MSCs, especially in nanofibrous form and when combined with matriline-3, a non-collagenous cartilage ECM protein (Liu et al., 2018).

Among scaffold-free techniques, cell sheet technology is used to develop transplantable constructs by stimulating MSC chondrogenic differentiation by spontaneously inducing post-detachment cell contraction leading to cytoskeletal reorganization (Thorp et al., 2020; 2021b). Moreover, multilayer cell sheets can be produced to increase 3D cellular interactions, especially those mediated by N-cadherin, connexin 43, and integrin β-1, enhancing *in vitro* chondrogenesis (Thorp et al., 2021a).

Another effective scaffold-free technique is 3D high-density culture for the manufacture of cartilage spheroids. These 3D-systems commonly display a hypoxic core that could promote chondrogenesis of stem cells. Moreover, interactions with adjacent cells simulate those found in pre-cartilage condensations during embryonic development (Ryu et al., 2019). These aggregates can be produced using a range of methods; however, the hanging drop technique allows to control 3D-system size by modifying drop volume or cell density (Ryu et al., 2019).

Despite 3D cultures are well-recognized methods to study chondrogenic potential of various tissue derived MSCs, the ability of these stem cells in a 3D setting to modulate chondrogenic differentiation and cytokine production has not been reported yet. Therefore, we investigated 3D high-density culture performance in promoting chondrogenic commitment of human stem cells, and in modification of cytokine expression during differentiation process. Specifically, BM and WJ-MSCs were used to produce 3D culture in the presence of hTGF-β1. Chondrogenic commitment was evaluated by gene expression profiling and immunohistochemistry (IHC) analysis of specific markers, such as types I, II, III, and X collagen, SOX9 transcription factor, and ACAN. Cytokine production was also monitored by pro- and anti-inflammatory cytokine gene expression and immunoassay along the differentiation events.

## Materials and methods

### hMSC isolation, expansion, and characterization

hBM-MSCs were obtained from bone marrow (BM) of three healthy donors (aged between 38 and 40); whereas hWJ-MSCs were isolated from human umbilical cord of three donors (age between 23 and 31). Donors gave written informed consent to the use of samples for research purposes, with approval of local Ethic Committee (Review Board prot./SCCE n. 24988). Briefly, BM aspirate or umbilical cord were seeded in Minimum Essential Medium Alpha (α-MEM) supplemented with 1% Glutagro™, 10% Fetal Bovine Serum (FBS), and 1% Penicillin/Streptomycin, and incubated at 37°C in an atmosphere of 5% CO_2_ and 95% relative humidity (Giordano et al., 2014). After 72 h, non-adherent cells and other residues were aspirated, fresh media added, and the remaining adherent cells fed with fresh media twice a week. On day 14, colonies of adherent MSCs were identified, detached, and re-seeded at 4,000 cells/cm^2^ in the same culture conditions. Once the cell culture reached 70–80% confluence, cells were detached using 0.05% trypsin-0.53 mM EDTA and washed with 1X phosphate buffered saline (PBS) (Corning Cellgro, Manassas, VA, United States), counted using Trypan Blue (Sigma-Aldrich, Milan, IT), and subcultured at a concentration of 4 × 10^3^ cells/cm^2^. At passage 2, cells were used for the experiments. Flow cytometry analysis was performed on both hBM-MSCs and hWJ-MSCs obtained at passage 2 with antibodies directed against CD90, CD105, CD73, CD14, CD34, CD45, and HLA-DR expression (Miltenyi Biotec B.V. & Co. KG, Bergisch Gladbach, Germania), as better described elsewhere (Ciardulli et al., 2020b, 2021; Scala et al., 2022).

### hMSC cultures

hBM-MSCs were seeded on coverslips at a concentration of 4 × 10^3^ cells/cm^2^. Once the cell cultures reached 60% confluence, cells were treated with chondrogenic medium and supplemented with either 1 ng/ml or 10 ng/ml of recombinant human TGF-β1 (PeproTech EC, Ltd., London, United Kingdom). The chondrogenic medium was composed of alpha-minimum essential medium (α-MEM) (Corning, NY, United States) with reduced FBS to 1% (Corning Cellgro) further supplemented with 1% ITS (Corning Cellgro), 1% GlutagroTM (Corning Cellgro), 50 µM of ascorbic acid phosphate, and 1% Penicillin-Streptomycin. Cells were fed twice a week with fresh medium and growth factor for up to 16 days. Untreated cells for all studied time-points were employed as control.

High density 3D cultures were obtained by the hanging drop method. Briefly, hMSCs were resuspended at a density of 5 × 10^5^ cells/mL. Drops of cell suspension (30 µl) were dispensed on the lids of Petri dishes, that were then inverted, and hanging drop cultures were incubated. After 3 days, resulting cellular aggregates were harvested using a pipette, and transferred into an ultra-low attachment 96 multi-well plate using 100 µL/well of chondrogenic medium (supplemented with 10 ng/ml of hTGF-β1), that was changed every 2 days. 3D systems morphology was monitored with ImageJ software (rel.1.52p National Institutes of Health, United States), and diameter, area (A) and perimeter (p) were measured, and circularity was calculated using Eqn. 1:

$$\text{F}\;\;\mathrm{circularity}=\frac{4\pi A}{p^{2}}$$ (1)

For the evaluation of Feret’s diameter and circularity, the average value of 10 3D-systems (*n* = 3), were considered (De Moor et al., 2020).

### RNA isolation and gene expression profiling

Type II collagen (*COL2A1*), SRY-Related HMG-BOX Gene 9 (*SOX9*), Aggrecan (*ACAN*), type I, III and X collagen (*COL1A1*, *COL3A1*, and *COL10A1*) markers were investigated, as well as pro-inflammatory cytokines Interleukin 6 (*IL-6*), Tumor Necrosis Factor α (*TNF-α*), Interleukin 12A (*IL-12A*), Interleukin 1β (*IL-1β*) and anti-inflammatory ones Interleukin 10 (*IL-10*), and *TGF-β1*. Total RNA was extracted from hBM-MSCs at each time point using QIAzol^®^ Lysis Reagent (Qiagen, DE), chloroform (Sigma-Aldrich) and the Rneasy Mini Kit (Qiagen, DE). For each sample, 1 μg of total RNA was reverse transcribed using the iScript™ cDNA synthesis kit (Bio-Rad, Milan, IT). Relative gene expression analysis was performed in a LightCycler^®^ 480 Instrument (Roche, IT), using the SsoAdvanced™ Universal SYBR^®^ Green Supermix (Bio-Rad, Foster City, CA, United States) with the validated primers (Bio-Rad) and following MIQE guidelines (Bustin et al., 2009). Amplification was performed in a 10 μl final volume, including 2 ng of complementary DNA (cDNA) as template. Triplicate experiments were performed for each explored condition, and data were normalized to glyceraldehyde-3-phosphate dehydrogenase (*GAPDH*) expression (Hellemans et al., 2007). Fold change in gene expression was determined by the 2^−ΔΔCt^ method and presented as relative levels vs. untreated cells at each time-point.

### Immunofluorescence assays

Cells were fixed with 3.7% paraformaldehyde (PFA) for 30 min at room temperature (RT), followed by permeabilization with 0.1% Triton × −-100 for 5 min and blocked with bovine serum albumin (BSA) solution (1% w/v) for 1 h. For type II and III collagen staining, cells were incubated overnight at 4°C with a rabbit polyclonal anti-type II collagen antibody (1:100; Cat no: ab34712, Abcam, Cambridge, United Kingdom) and a mouse polyclonal anti-type III collagen antibody (1:100; Cat no: sc166316, Santa Cruz Biotech., CA, United States). Then, cells were incubated for 1 h at RT with the Alexa Fluor ™ 488 goat anti-rabbit IgG (1:400; Thermo Fisher Scientific, Waltham, MA, United States) and the DyLight 649 anti-mouse IgG (1:500; BioLegend, San Diego, CA, United States) antibody. Cell nuclei were counterstained with 4′,6-diamidino-2-phenylindole (DAPI). Images were acquired using Leica laser-scanning confocal microscope (mod. TCS SP5; Leica Microsystems, Wetzlar DE) equipped with a plan Apo 63X/1.4 NA oil immersion objective. Signal intensity, related to the proteins of interest, was quantified using ImageJ software (rel.1.52p National Institutes of Health, United States) (Suchorska et al., 2016), when reported. Five images of several fields were used for the analysis at each time point. All data were reported as fold change relative to untreated cells. Antibody specificity was assessed in our previous works (Ciardulli et al., 2020c, 2021; Lamparelli et al., 2021).

3D-spheroids were fixed in 4% PFA for 2 h at room temperature, cryo-protected in 30% sucrose (4°C, overnight), included, and sliced in 15 μm thickness samples using a cryostat (mod. CM 1950, Leica, Wetzlar, Germany). For type I and II collagen staining, samples were incubated overnight at 4°C with a mouse polyclonal anti-collagen type I antibody (1:100, Cat no: MAB3391, Abcam) and rabbit polyclonal anti-collagen type II antibody (1:100, Cat no: ab34712, Abcam). Subsequently, slices were incubated for 1 h at RT with Alexa Fluor ™ 488 goat antirabbit IgG (1:400; Thermo Fisher Scientific) and DyLight 649 anti-mouse IgG (1:500; BioLegend) antibodies. Cell nuclei were counterstained with DAPI.

### Sirius red staining

For cartilage matrix histochemistry, a Sirius Red staining was performed as previously described (Hyllested et al., 2002; Greiner et al., 2021). 3D-spheroid sections were stained in hematoxylin for 8 min, washed in water for 2 min, immersed into phosphomolybdic acid for 2 min, and washed in water for 2 min. Subsequently, samples were dipped into Picrosirius Red F3BA Stain (Polysciences, Inc., Warrington, PA, United States) for 60 min, and finally into HCl 0.1 M solution for 2 min. Samples were dehydrated with an increasing ethanol gradient (70%–75%–95%–100%) and cleared in xylene for 5 min. Sections were then mounted using Eukitt (Sigma-Aldrich) mounting medium. Picrosirius red brightfield and polarized light images were acquired with a Brunel polarization microscope equipped with a Nikon D500 camera.

### Safranin-O staining

Spheroids were fixed in 4% paraformaldehyde (PFA) for 24 h at room temperature, cryo-protected in 30% sucrose (4°C, overnight), included in optimal cutting temperature (OCT) compound, and cut in 10 μm-thick slices through a cryostat microtome (mod. CM 1950, Leica, Wetzlar, Germany). Safranin-O staining was used to detect acidic proteoglycans present in the ECM, according to standard methods (Schmitz et al., 2010b). In more detail, slices were stained for 10 min with Wiegert’s Iron Hematoxylin (Biognost), 5 min with 0.5 g/L Fast green (Sigma-Aldrich), and 7 min with 0.1% Safranin-O solution (Merck Millipore, United States). Samples were dehydrated with an increasing ethanol gradient (70%–75%–95%–100%) and cleared in xylene for 2 min. Sections were then mounted using Eukitt (Sigma-Aldrich) mounting medium. Safranin-O brightfield images were acquired with a microscope (BX53, Olympus, Tokyo, Japan) equipped with an Olympus SC180 camera (Tokyo, Japan) and Olympus U-TV0.5XC-3 camera adapter operating Olympus cellSens standard 3.2 software.

### Live and dead staining

Cell viability within scaffolds was detected by fluorescence live/dead assay (Calcein AM solution; Cat. no C1359) and Ethidium homodimer I solution (Sigma-Aldrich). In more detail, cells were stained for 1 h at 37°C, washed in 1 × PBS, and captured by fluorescence microscope (mod. Eclipse, Nikon Corporation, Tokyo, Japan). Single images were acquired with identical light intensity, exposure time, and gain settings. Signals intensity was quantified using ImageJ software (rel.1.52p National Institutes of Health, Bethesda, MD, United States). Original images in RGB format were converted into an 8-bit (gray scale) format, and tagged areas were expressed as an average value of pixel intensity within a range from 0 (dark) to 255 (white) as better described elsewhere (Spaepen et al., 2011).

### Bead-based multiplex immunoassay

For cytokine measurement in culture medium, a bead-based multiplex immunoassay was employed with two sets of beads (Beads A and Beads B) using an internal fluorescence intensity detected in APC channel. A 9-plex LEGENDplex™ Cutom Panel (BioLegend) was designed to measure IL-1β, IL-6, TNF-α, hepatocyte growth factor (HGF), IL-15, IL-10, macrophage inflammatory protein (MIP)-1α and 1β, and Dickkopf-related protein 1 (DKK1). A calibration curve was prepared for cytokine quantification following manufacturer’s instructions. Samples were diluted 1:50 with fresh complete α-MEM medium and run in duplicate. Specimens were acquired on a BD FACSVerse cytometer (BD Biosciences) equipped with two lasers (blue, 488 nm; and red lasers, 628 nm) and BD FACSuite software (BD Biosciences), and at least 3,400 beads were recorded. LEGENDplex™ Data Analysis Software Suite (BioLegend) was used for post-acquisition analysis.

### Statistical analysis

For comparison between two independent groups, two-tailed independent Student’s *t* test was performed. One-way or two-way ANOVA was used, followed by Tukey’s multiple comparison test for comparisons between more than two groups. *p* values < 0.05 were accepted as significant (de Winter 2013).

## Results

### hTGF-β1 at 10 ng/ml efficiently induces chondrogenic differentiation of hBM-MSCs

**FIGURE 1:**
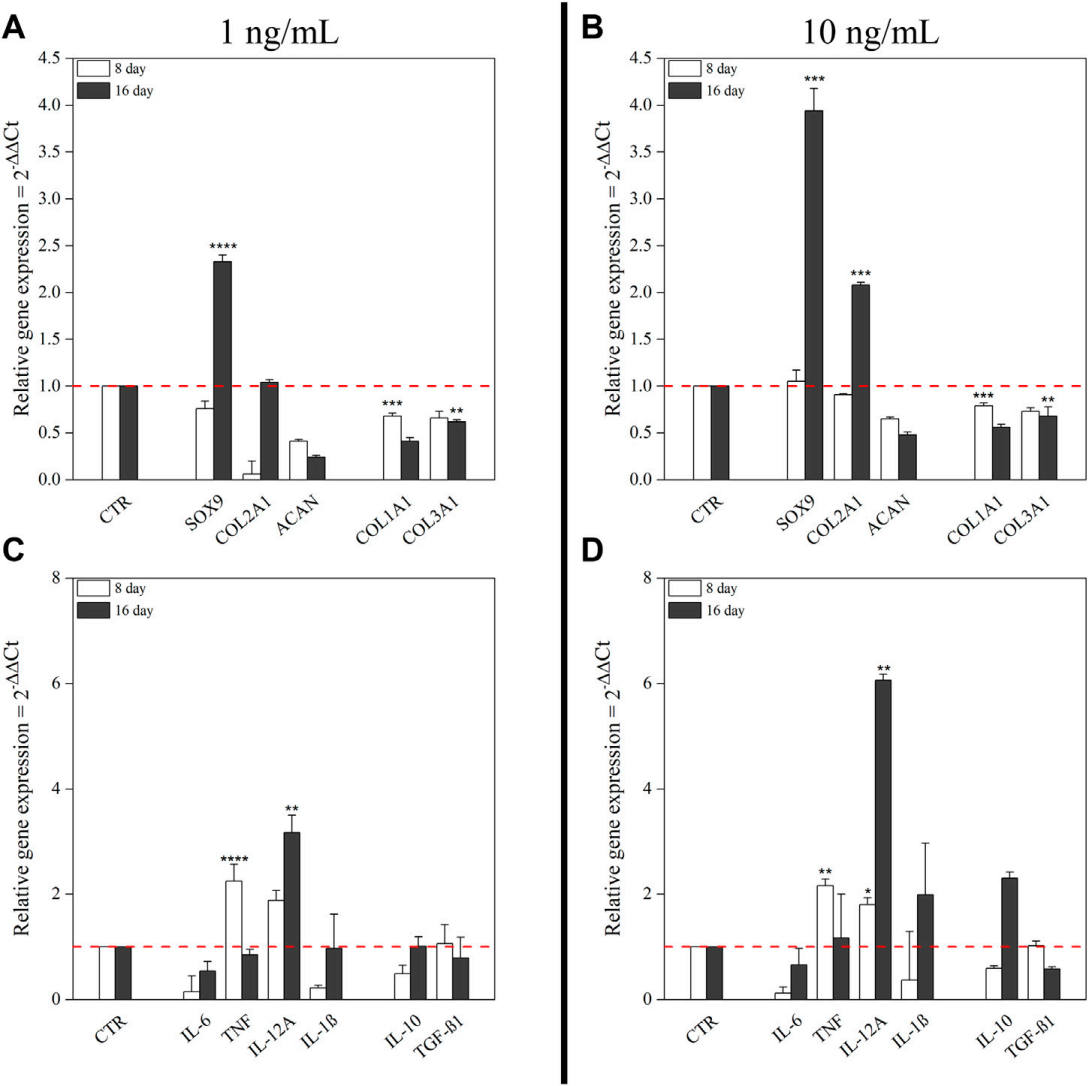
Effect of hTGF-β1 on the expression of chondrogenic markers and cytokines by hBM-MSC monolayer. Two different concentrations of hTGF-β1 (1 ng/ml and 10 ng/ml) for up to 16 days were tested. Untreated cells at matched time points were used as control. mRNA expression levels of positive and negative chondrogenic markers (*COL1A1*, *COL2A1*, *COL3A1*, *SOX9*, and *ACAN*) at 1 ng/ml **(A)** and at 10 ng/ml **(B)**; pro- and anti-inflammatory cytokines (*IL-6*, *TNF-α*, *IL-12A*, *IL-1β*, *IL-10*, and *TGF-β1*) at 1 ng/ml **(C)** and at 10 ng/ml **(D)**. All data were analyzed by two-way ANOVA, *N* = 3 (biological replicates); *p < 0.05, **p < 0.01, ***p < 0.001, and ****p < 0.0001 vs. control.

To identify optimal conditions for chondrogenic commitment of hBM-MSC, a medium supplemented with two concentrations of hTGF-β1 (1 and 10 ng/ml) was tested in a 16-day culture system. Higher growth factor concentrations were not considered, because published literature describes inhibitory effects (Huang et al., 2004b). Unsupplemented cells harvested at matched time points were used as negative control. Culture medium with 1 ng/ml hTGF-β1 resulted in a 2.33-fold increase (*p* < 0.0001) of *SOX9* levels at day 16, whereas 10 ng/ml hTGF-β1 stimulated a *SOX9* 3.94-fold up-regulation (*p* < 0.001). The lower hTGF-β1 supplementation induced a marked *COL2A1* downregulation at day 8 followed by expression recovery to control levels at day 16. In contrast, at 10 ng/ml concentration, *COL2A1* expression showed a 2-fold increase only at day 16. *ACAN* was not detected at each time point at both concentrations explored. *COL1A1* and *COL3A1* expression were always downregulated in response to hTGF-β1 supplementation (Figures 1A,B).

Cytokines expression along chondrogenic events was also monitored. Supplementation with 1 ng/ml hTGF-β1 resulted in transcriptional upregulation of pro-inflammatory cytokines, especially *TNF-α* and *IL-12A*, respectively 2.25-fold (*p* < 0.0001) and 1.88-fold, at day 8; while no changes in anti-inflammatory cytokines were observed. After 16 days, only *IL-12A* retained significant upregulation (3.17-fold; *p* < 0.01) (Figure 1C). At 10 ng/ml, hTGF-β1 supplementation promoted *TNF-α* (2.16-fold; *p* < 0.01) and *IL-12A* (1.8-fold; *p* < 0.05) upregulation after 8 days. Moreover, *IL-12A* (6.06-fold; *p* < 0.01) and *IL-1β* (1.99-fold) displayed significant changes at day 16. Among anti-inflammatory cytokines, only *IL-10* was upregulated (2.3-fold) (Figure 1D).

**FIGURE 2:**
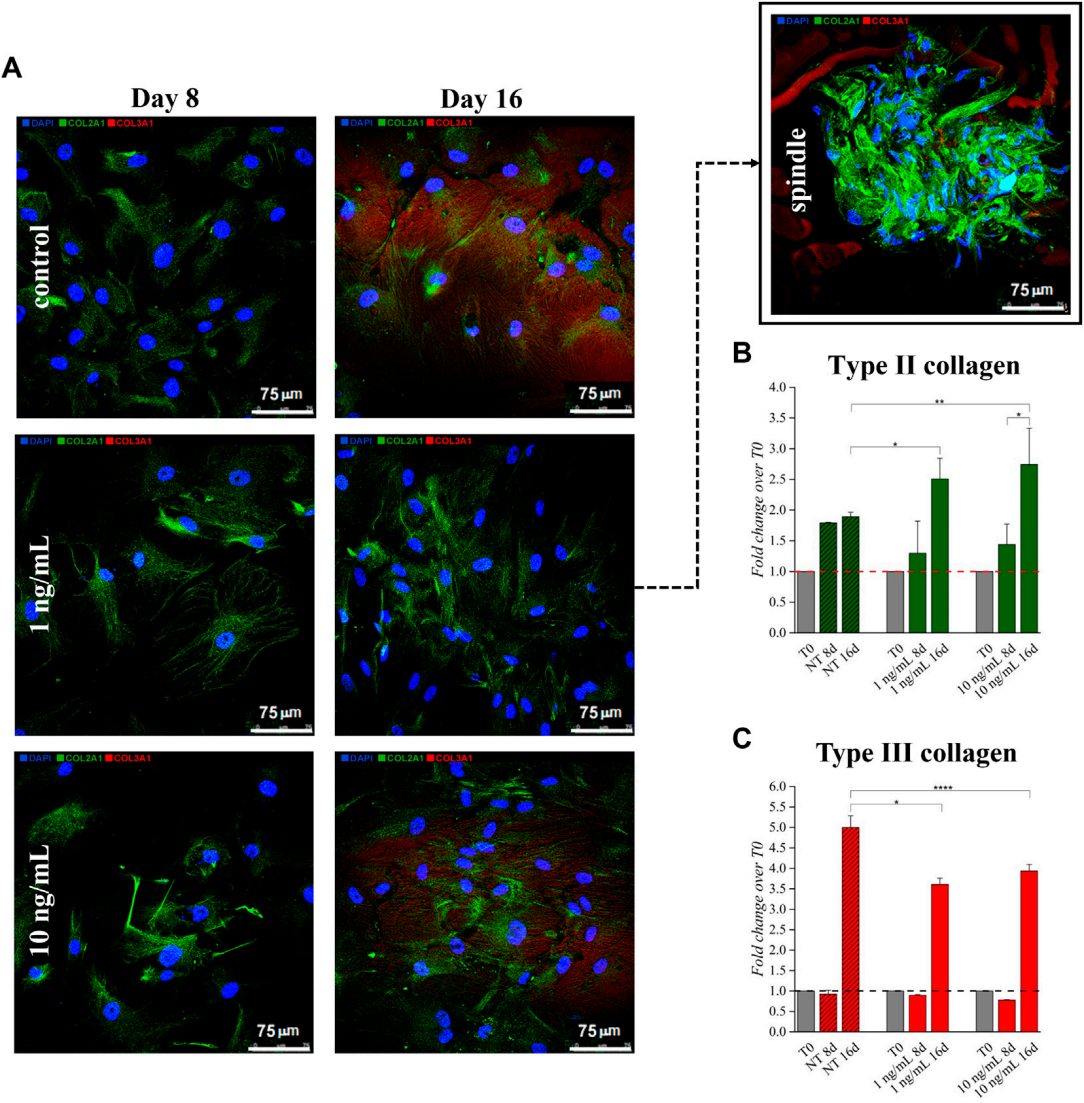
Effect of hTGF-β1 on the expression of type II (stained in green) and type III (stained in red) collagens by BM-MSC monolayer. Spindle-shaped systems were observed at Day 16 only when 1 ng/ml hTGF-β1 was supplemented **(A)**. Higher type I collagen stain was observed when 10 ng/ml were supplemented, as indicated by signals relative quantification **(B,C)** *p < 0.05, **p < 0.01, ***p < 0.001, and ****p < 0.0001, one-way ANOVA. *N* = 3 (biological replicates). Scale bar: 75 µm.

Effects of hTGF-β1 on type II and III collagen production was monitored by semi-quantitative immunofluorescence (q-IF) assay (Figure 2A). hBM-MSCs displayed low basal expression of types II and III collagen that increased following 16 days of continuous hTGF-β1 exposure; however, type II collagen staining (green) was more intense when cells were supplemented with 10 ng/ml of growth factor at day 16. This behavior was confirmed by q-IF data, which indicated an increase (2.74-fold) of the stained protein signal at day 16 (Figure 2B). Supplementing 1 ng/ml, several spindle shaped aggregates were observed at day 16. No spindle systems were evidenced when a concentration of 10 ng/ml was used. Type III collagen signal was upregulated at day 16 in all conditions when compared to baseline (Figure 2C), while its intensity decreased following chondrogenic medium treatment compared to matched untreated cells.

Based on these data, 10 ng/ml hTGF-β1 was chosen as the optimal concentration for culture medium supplementation in the next 3D high-density approach.

### 3D high-density culture

**FIGURE 3:**
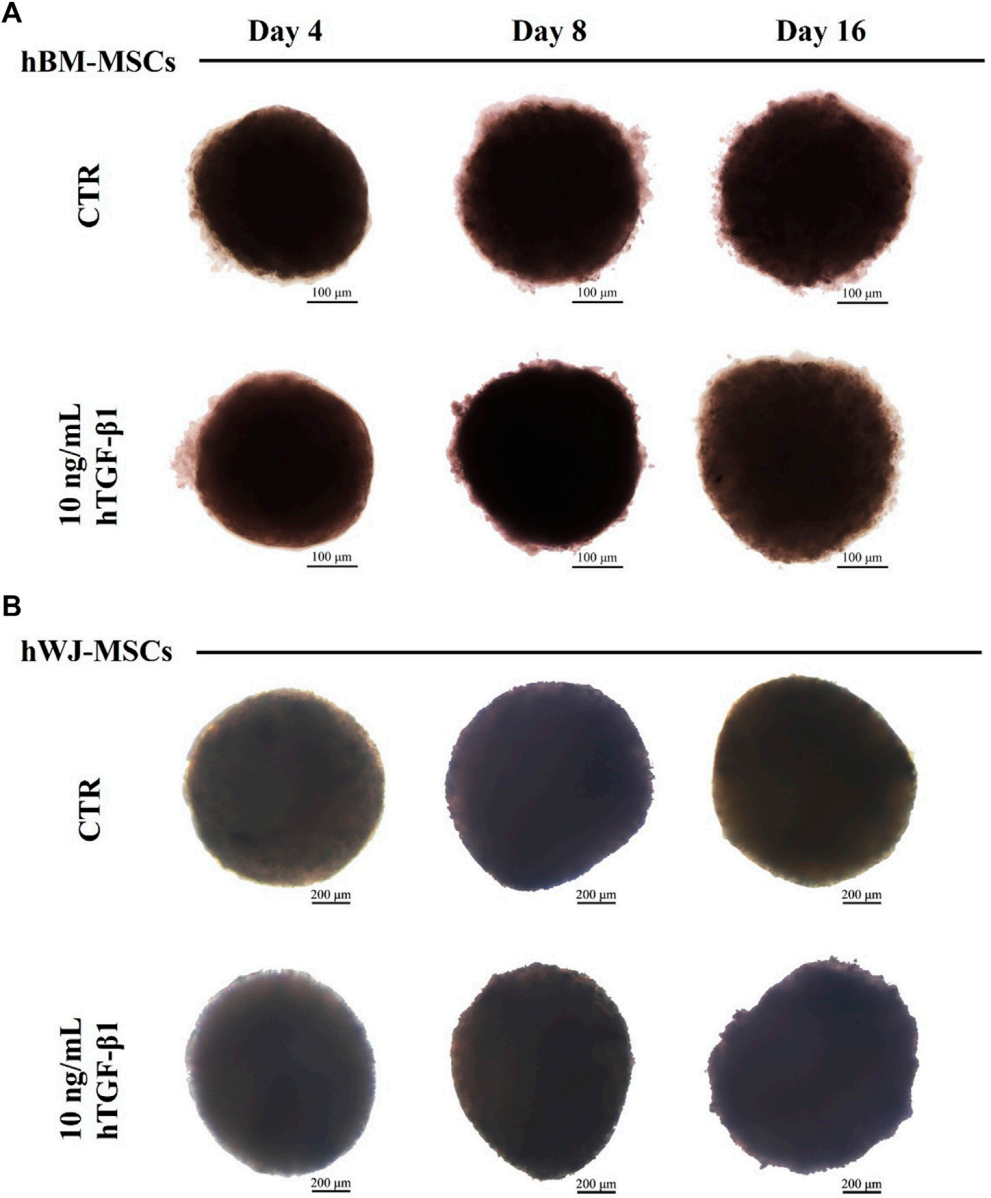
Brightfield images of hBM-MSCs **(A)** and hWJ-MSCs **(B)** 3D high-density culture supplemented with 10 ng/ml of hTGF-β1 and acquired at Days 4, 8, and 16. Untreated spheroids (CTR) at matched time-points were presented for control purposes. Scale bars were 200 micron for BM culture and 100 micron for WJ one.

3D high density culture were monitored for 16 days of incubation using brightfield microscopy for morphological analysis. hBM-MSC showed spherical structures with smooth borders, especially after 4 and 8 days (Figure 3A). A mean Feret’s diameter of 450 µm (±64) and circularity of 0.54 µm (±0.16) were calculated after 16 days of treatment with hTGF-β1. Similarly, control 3D culture without growth factor had a mean diameter of 460 µm (±64), with a lower circularity of roughly 0.46 µm (±0.11). hWJ-MSC 3D systems were bigger and more regular, with a mean Feret’s diameter of 1,163 µm (±69) and circularity of 0.62 µm (±0.08) at the end of chondrogenic culture (Figure 3B).

**FIGURE 4:**
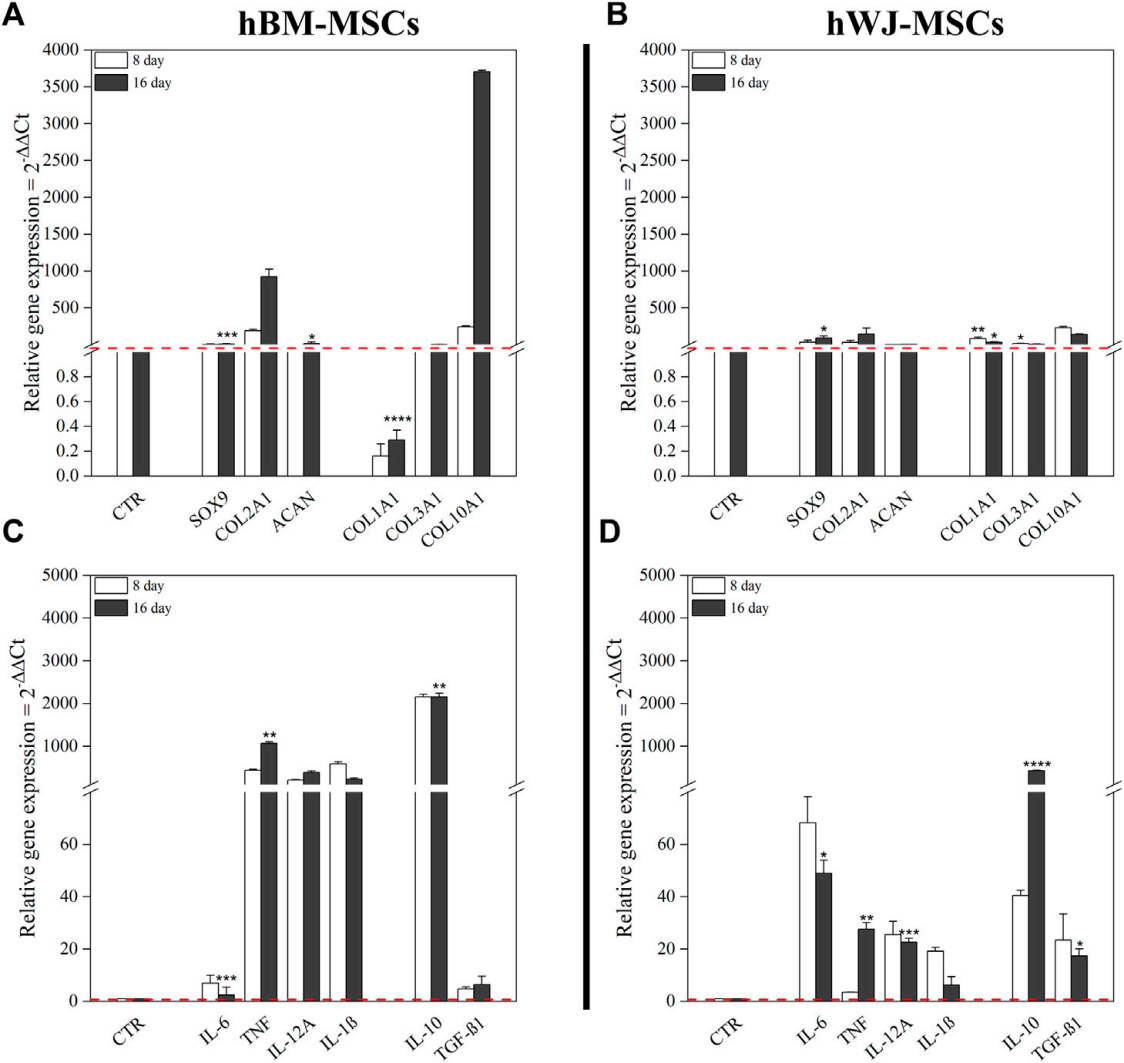
Gene expression profiles of both hBM-MSCs and hWJ-MSCs based high density culture supplemented with 10 ng/ml of hTGF-β1. mRNA expression levels of positive and negative chondrogenic markers (*COL1A1*, *COL2A1*, *COL3A1*, COL10A1, *SOX9*, and *ACAN*) **(A,B)** and cytokines (*IL-6*, *TNF-α*, *IL-12A*, *IL-1β*, *IL-10*, and *TGF-β1*) **(C,D)**; untreated cells at time zero were used as control. *p < 0.05, **p < 0.01, ***p < 0.001, and ****p < 0.0001 (two-way ANOVA). *N* = 3 (Biological replicates).

Gene expression profiles of chondrogenic markers and cytokines were monitored along the experimental timeline. All chondrogenic markers were upregulated in 3D culture combined with hTGF-β1 treatment. At day 8, hBM-MSCs displayed upregulation of *SOX9* (3.15-fold), *COL2A1* (191.68-fold), *ACAN* (1.50-fold), *COL3A1* (1.13-fold), and *COL10A1* (242.2-fold). At day 16, increased upregulation was noted for *SOX9* (8.05-fold, *p* < 0.001), *COL2A1* (925.44-fold), *ACAN* (15.16-fold), and *COL10A1* (3704-fold), whilst *COL1A1* was downregulated and *COL3A1* was unchanged compared to control (Figure 4A). On the other hand, WJ-3D systems at day 8 displayed strong upregulation of *SOX9* (33.04-fold), *COL2A1* (30.76-fold), *COL1A1* (83.55-fold), *COL3A1* (13.94-fold), and *COL10A1* (231.5-fold) while *ACAN* underwent slight upregulation. At day 16, *SOX9* (89.30-fold, *p* < 0.001), *COL2A1* (146.60-fold), and *ACAN* (4.27-fold, *p* < 0.05) all underwent further upregulation, while *COL1A1* (29.94-fold, *p* < 0.001), *COL3A1* (6.39-fold) and *COL10A1* (137.6-fold) displayed downregulation in comparison to day 8 levels (Figure 4B).

**FIGURE 5:**
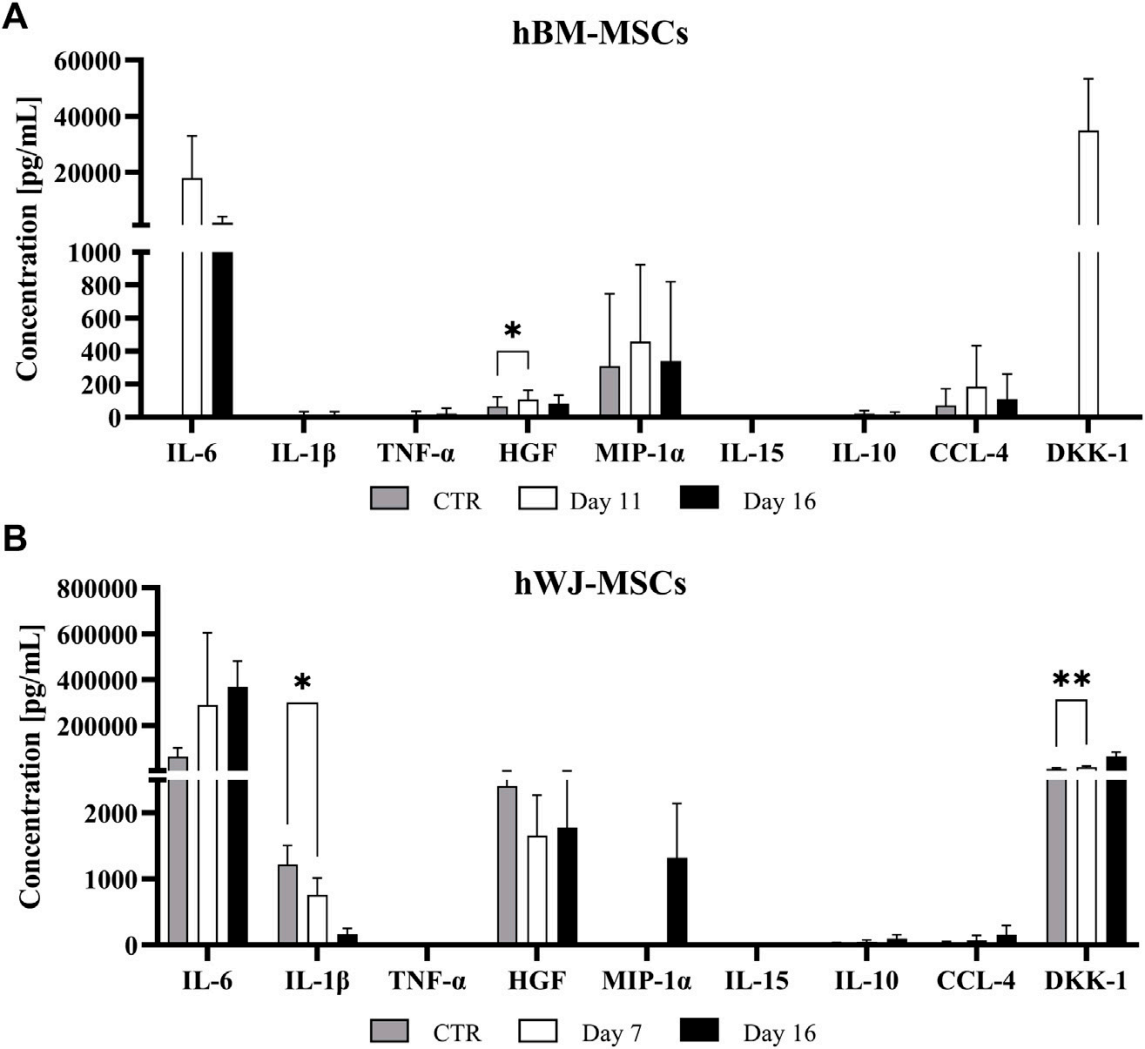
Cytokine release profiles of BM **(A)** and WJ **(B)** 3D culture in a chondrogenic medium supplemented with 10 ng/ml of TGF-β1 for up to 16 days. Cytokine levels expresses as (pg/ml) were measured in the culture medium using a multiplex bead-based immunoassay at various time points and shown as means ± SD. *p < 0.05 and **p < 0.01 (*N* = 2).

hBM-MSC upregulated all pro-inflammatory cytokines genes (e.g., *TNF*-α and *IL-12A*, respectively 440.75-fold and 205-fold, at day 8). Among anti-inflammatory cytokines, *IL-10* showed a strong upregulation with a 2154-fold change. After 16 days, only *TNF-α* underwent a further increase (1071-fold, *p* < 0.01), whereas the other ones retained an expression similar to that of previous time-points. Moreover, at the end of culture, *IL-10* maintained its upregulated (2158-fold, *p* < 0.01), instead, *TGF-β1* retained a slight increase compared to control (Figure 4C). However, at protein level, IL-6 tended to increase in the culture medium from day 11, as well as MIP-1α, CCL-4, and DKK1, while only HGF was significantly higher at day 11 compared to baseline (*p* = 0.0136) (Figure 5A). Other cytokines were not detected. In hWJ-MSCs, cytokine expression resulted in limited transcriptional upregulation of all pro-inflammatory cytokines genes, with *IL-6* that reached the highest fold-value (68.24-fold) at day 8, while *IL-10* showed a strong upregulation (40.47-fold). As the culture progressed, *TNF-α* (27.65-fold, *p* < 0.01) and *IL-12A* (22.61-fold, *p* < 0.001) significantly increased, while other pro-inflammatory cytokines displayed a lower shift. Moreover, *IL-10* and *TGF-β1* expression increased compared to control, reaching respectively 430-fold (*p* < 0.0001) and 17.31-fold (*p* < 0.05) (Figure 4D). At protein level, IL-6 tended to increase in the culture medium from day 7, as observed in BM-MSC cultures, while IL-1β was significantly reduced starting from day 7 (*p* = 0.0265) and DKK1 increased at day 7 (*p* = 0.0015) compared to baseline (Figure 5B).

**FIGURE 6:**
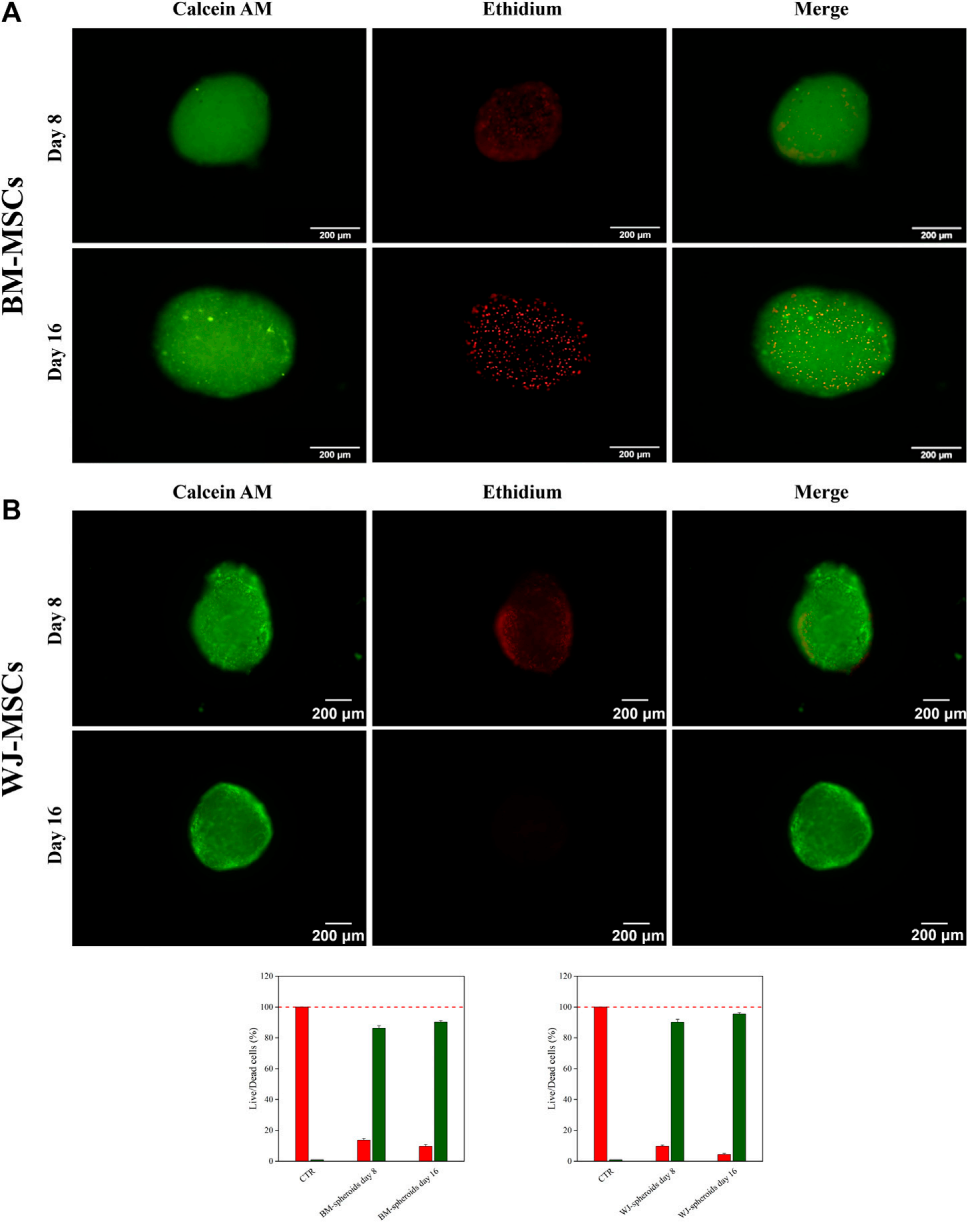
Live & Dead assay and quantify fluorescent signal of hBM-MSCs **(A)** and hWJ-MSCs **(B)** in 3D high-density cultures supplemented with 10 ng/ml of hTGF-β1 and acquired at Days 8 and 16. Viable cells appear green, non-viable cells in red. Scale bar: 200 µm.

Cell viability inside 3D aggregate systems was performed after 8 and 16 days of culture using Live and Dead assays, and micrographs are shown in Figure 6A-B for hBM-MSCs and WJ-MSCs, respectively. The red signal, associated with dead cells, was detectable only in the core of 3D systems and varied from 5% at day 8–10% at day 16. Therefore, cultures longer than 16 days were not considered for both cells type, as also suggested by literature (Lee et al., 2017).

**FIGURE 7:**
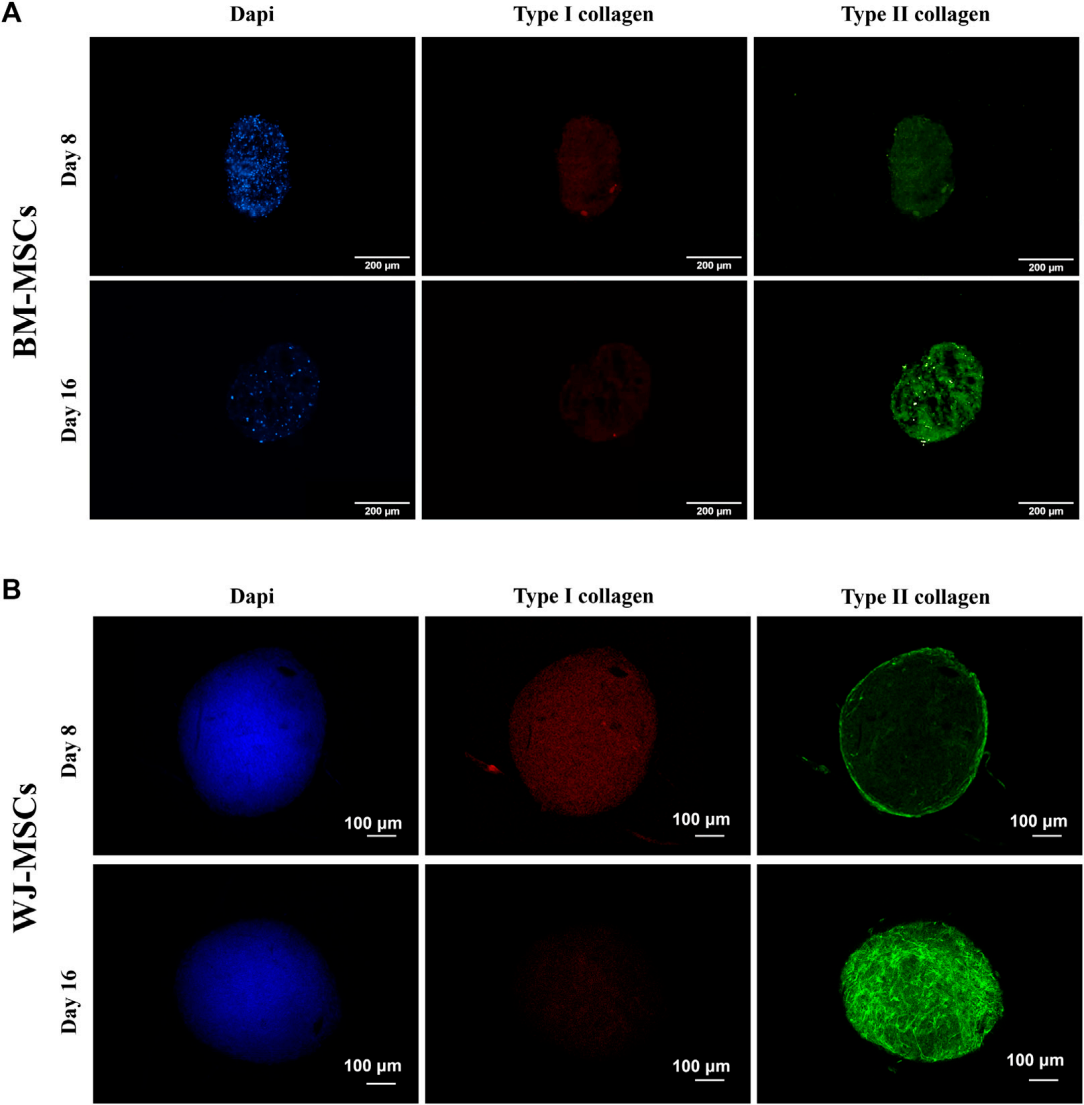
Immunofluorescence of hBM-MSCs **(A)** and hWJ-MSCs **(B)** in 3D high-density cultures supplemented with 10 ng/ml of hTGF-β1 and acquired at different time-point. Type I collagen was stained in red and type II collagen in green. DAPI was used to counterstain the nuclei (blue) Scale bars were 200 microns for BM culture and 100 microns for WJ one.

**FIGURE 8:**
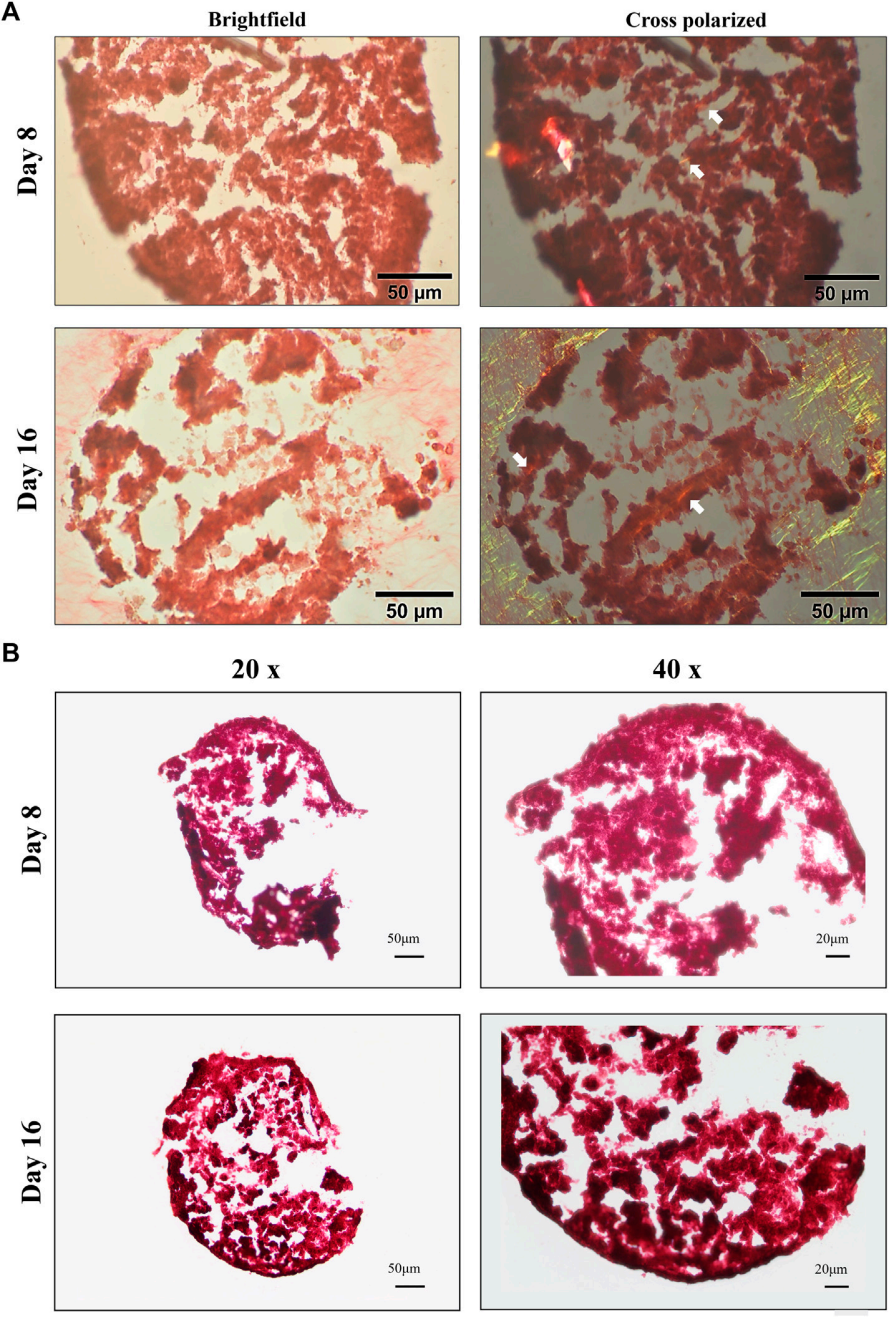
Brightfield and cross-polarized images of picrosirius red **(A)** and Safranin-O **(B)** staining on 3D high density culture at days 8 and 16. Fine birefringent collagen fibers were visible by day 8 and day 16 (see white arrows) in BM culture. Scale bar: 50 µm **(A)**; accumulation of acid proteoglycans (stained in red) after 8 and 16 days in WJ-3D culture. Scale bar 20 µm **(B)**.

Production of type I and II collagen (proteins) was monitored by immunofluorescence (IF) assay (see Figures 7A,B). BM-MSC 3D-system displayed a basal expression of type I collagen, that decreased following 16 days of continuous hTGF-β1 exposure; however, type II collagen staining (green) was more intense at day 16. The same trend was observed for WJ-MSC 3D system as documented by IF micrographs, where the green signal associated with type II collagen was markedly increased from 8 to 16 days. Histological evaluation by Sirius red acquired with a polarized microscope to intercept the birefringence of collagen fibers was also performed on both 3D culture. An example of the intensity of red signal, mainly associated with type II collagen, increased from 8 to 16 days, confirming previous IF data. Translucent new fibers were also clearly detected at day 16 of culture (Figure 8A). Further histological evaluation by Safranin-O for WJ-3D culture was performed and detected the accumulation of acid proteoglycans (Figure 8B).

## Discussion

3D *in vitro* chondrogenic models using human stem cells derived from different tissues have great potential in cartilage healing and regeneration (Mobasheri et al., 2014). In this study, we investigated chondrogenic propensity of human MSCs harvested from BM and WJ using 3D high-density culture coupled with hTGF-β1 supplementation. We also observed the variations in cytokine production during differentiation by gene expression profiling for pro- and anti-inflammatory cytokine genes.

Chondrogenesis from human MSCs is a complex process, involving the activation of different genes, especially the transcription factor *SOX9* that plays a key role in trigging the differentiation phase. Indeed, its upregulation promotes stem cell commitment toward chondrogenic phenotype and reduces hypertrophic drift leading to cartilage mineralization, often accompanied by apoptosis of chondrocytes and matrix ossification (Mueller and Tuan, 2008; Jiang et al., 2018). While *SOX9* is rapidly upregulated along chondrogenesis, the expression of other genes emerges later. *COL2A1* represents the gold standard among chondrogenic markers and is usually detectable only after several days of chondrogenic induction. In preliminary monolayer experiments, chondrogenesis-related gene expression was not affected by 1 ng/ml hTGF-β1 supplementation in culture medium, while increased when using 10 ng/ml after 16 days of culture, in agreement with previously published data (Li et al., 2011). *ACAN*, another chondrogenic marker, was not detected at any time point at 1 and 10 ng/ml hTGF-β1 concentrations, possibly because its expression becomes evident between 18 and 24 days of culture (Xu et al., 2008); however, some variations are described due to biological variabilities, number of culture passages, different media and TGF (β1 or β3) used, and cell types employed (e.g., adipose-, BM-, or WJ-derived MSCs) (Sacchetti et al., 2016; Huynh et al., 2019; Futrega et al., 2021). Higher concentrations of hTGF-β1 have already been reported to exert inhibitory effects on adipose-derived MSCs (Huang et al., 2004a). Therefore, we did not test higher doses in our monolayer experiments and we chose 10 ng/ml of hTGF- β1 for the 3D culture systems as the optimal condition because of a significant increase in chondrogenic-related genes. Conversely, *COL1A1*, *COL3A1*, and *COL10A1* were employed as negative markers because largely reported to be dedifferentiation genes of hyaline cartilage phenotype and often associated with differentiation towards other musculoskeletal lineages. Type I collagen gene expression is related to both tenogenic commitments (Ciardulli et al., 2020b) and fibrocartilage tissue (Kovermann et al., 2019). Moreover, its expression increases in chondrocytes during progression of human osteoarthritis (Zhong et al., 2016). Type III collagen is often co-localized with type I within the same fibril (Dehne et al., 2010), for this reason, it is also considered as a negative marker. However, its exact role in chondrogenesis remains unclear because contrasting evidence on its protective or pathological functions after injuries is reported (Alcaide-Ruggiero et al., 2021). The development of a stellate or fibroblast-like phenotype during culture compromises clinical outcome of potential regenerative therapy and is a major issue for application of hBM-MSCs in cartilage defect repair (Li et al., 2019). According to published literature, these genes were downregulated in response to 1 and 10 ng/ml hTGF-β1 concentrations in our monolayer experiments confirming chondrogenic commitment of stem cells in our system culture.

*In vitro* chondrogenic BM-MSC differentiation in 3D high-density cultures supplemented with TGF-β1 or TGF-β3 is often accompanied by up-regulation of genes associated with chondrocyte hypertrophy, including type X collagen and its transcription factor (RUNX-2), or matrix metalloproteinases (MMPs), and by activation of alkaline phosphatase (ALP) (Agar et al., 2011). Hypertrophic processes are strongly dependent on types and concentrations of chondrogenic inducers used and also on *in vitro* culture time (Mueller et al., 2010). In our study, the early phase of chondrogenic differentiation, also known as pre-differentiation or commitment phase, was investigated using a short culture period (only 16 days). Even though our system could not prevent hypertrophic drift of BM-derived MSCs, we observed a high chondrogenic potential not only in the case of BM- but also with WJ-derived stem cells in 3D high-density conditions and we also documented *COL10A1* downregulation over time (from 8 to 16 days) for WJ-culture, thus showing a less tendency to hypertrophic drift of these cells. WJ-MSCs are another possible source of mesenchymal stromal cells for regenerative medicine approaches; however, few or absent data are present about hWJ-MSC chondrogenic commitment efficiency. In our study, we assumed that similar events to those occurring in hBM-MSCs may be induced in WJ cells. Similar to that reported in 2D BM-MSC culture with 10 ng/ml of TGF-β1, WJ-MSCs showed *SOX9* upregulation at day 16, and *COL1A1* decreased expression from day 8–16. These results support the use of WJ-MSCs as a valid alternative for chondrogenic commitment of stem cells in regenerative medicine.

Monolayer culture systems have various limitations, especially in chondrogenic differentiation of human stem cells, because cells grow in adhesion at the flask bottom without mimicking the complex physiological *in vivo* tissue architecture. When stem cells are cultured in 2D tend to lose their stemness and differentiation potential (Lei and Schaffer, 2013), as documented by the lack of *ACAN* expression due to the short culture period in order to avoid de-differentiation processes on long-term 2D chondrocyte cultures (Caron et al., 2012b). Moreover, living 3D structures seem to be advantageous, especially in chondrogenesis studies, due to the presence of a hypoxic core within the structure that could stimulate differentiation. Indeed, recent studies have confirmed that hypoxia is a full-fledged chondrogenic stimulating factor, because articular cartilage is an avascular tissue with a reduced oxygen and nutrient intake. Hypoxia seems to better reproduce the natural chondrocyte environment (Lee et al., 2013; Citeroni et al., 2021). In our study, successful 3D high-density cultures were obtained when 5 × 10^5^ cells/ml were used. BM-MSC 3D systems with a mean Feret’s diameter of 401 ± 39 µm and circularity of 0.60 ± 0.14 µm were obtained after 4 days of culture with 10 ng/ml hTGF-β1 supplemented medium. As culture moved forward, these systems became larger in size, but less regular in shape at day 16, probably due to the proliferative effect of TGF-β1 (Giudice et al., 2021). Lower cell amounts resulted in smaller and frailer 3D systems making extremely challenging further studies (data not shown).

BM-MSC 3D system was more effective than 2D cultures in driving chondrogenesis, as confirmed by the higher upregulation of markers associated with hyaline cartilage, including *ACAN* at day 16, not observed in monolayer experiments. Moreover, *COL1A1* was significantly downregulated at day 16, confirming the capability of the 3D system to inhibit the expression of this gene (Danišovič et al., 2012b). In contrast, hWJ-MSCs high-density culture showed larger and more regular aggregates, with a mean Feret’s diameter of 1,113 ± 28 µm and circularity of 0.73 ± 0.08 at day 4 and reaching values of 1,163 ± 69 μm and 0.62 ± 0.08 µm, respectively in diameter and circularity at the end of chondrogenic culture, thus showing a rise in size trend but a slight decrease in circularity over time. Different 3D culture morphology between BM and WJ-MSCs could be caused by various patchy adhesion forces and a higher proliferative rate of WJ-MSCs compared to BM-MSCs. Indeed, cell aggregates formation can be influenced by different parameters, such as cell suspension density, manufacturing method, medium composition, or incubation time. Cells suspensions assemble into 3D pellets because of cell-to-matrix and cell-to-cell interactions via integrins and cadherins (Foty and Steinberg, 2005; Gionet-Gonzales and Leach, 2018). Despite their larger diameters, WJ-3D cultures overexpressed all chondrogenic markers, including *ACAN* at day 16 and *COL2A1*, with marked downregulation of *COL1A1* and *COL3A1* already starting from day 8 through day 16, suggesting that also WJ-3D system can be effective *in vitro* tool for cartilage tissue engineering.

3D system also displayed good cell viability, even if small mortality was observed within the core. This behavior could be due to hypoxic stress or low nutrient exchanges to which the cells within the system are subjected. Looking at immunofluorescence data of the 2D culture, up to 3-fold change of collagen II was measured by q-IF at day 16, coupled with a slight downregulation of collagen III along the culture (1-fold), especially when 10 ng were supplemented. A further observation must be done, when supplementing 1 ng/ml in BM-2D culture; i.e., in this condition spindle-shaped aggregates were detected only at Day 16; these spindle structures may be considered an early organization of cellular 3D structure in the context of tenogenic commitment (Barboni et al., 2012). Therefore, 1 ng/ml hTGF-β1 dose may stimulate alternative differentiation pathways, while 10 ng/ml hTGF-β1 could drive hBM-MSC chondrogenic commitment (Puetzer et al., 2010).

MSCs can have immunomodulatory activities as described in several hematological disorders, such as primary myelofibrosis where they can also promote marrow fibrosis, or acquired aplastic anemia where BM-MSCs maintain the physiological balance between T regulatory cells and T helper (Th) 17 lymphocytes in the BM niche (Huo et al., 2020). Moreover, cytokines are important in regulation of normal chondrogenesis and pathological drift, as during osteoarthritis (Zhou et al., 2018). However, modifications of cytokine expression during chondrogenic commitment in both 2D and 3D culture systems using both BM- and WJ-derived MSCs have not been investigated yet. In our study, we demonstrated that when supplementing 10 ng/ml of hTGF-β1 in 2D system, TNF-α and IL-12A, two pro-inflammatory cytokines, were upregulated starting from day 8 of culture. TNF-α and IL-1β might hamper chondrogenesis of mesenchymal stromal cells, through NF-κB pathway, inhibition of SOX9 expression, and increase the synthesis of degradative enzymes involved in cartilage degeneration, like matrix metalloproteinases (MMPs) (Jagielski et al., 2014). Other studies suggest that *in vitro* exposure to TNF-α might increase proliferation, migration, and osteogenic differentiation of MSCs (Voskamp et al., 2020). In our system, BM- or WJ-3D culture showed an upregulation of all pro-inflammatory cytokine genes explored at day 8, except for IL-6 that showed a poor upregulation followed by a decrease at day 16, as also observed at the protein levels by measuring cytokines in culture medium. IL-6 behavior is in agreement with a previous study, where its expression during chondrogenic differentiation by pellet culture decreases from day 8 to day 16 (Kondo et al., 2015). During chondrogenic commitment, the most expressed cytokine was IL-10, as we also have observed (Behrendt et al., 2018); however, this cytokine was undetectable in culture medium. Therefore, both BM- and WJ-derived MSCs can produce pro-inflammatory cytokines that might support chondrogenic processes in an autocrine manner, as proposed by more pronounced variations in chondrogenic-related genes in 3D systems. Indeed, cell-to-cell contacts and spheroid structure in 3D culture better resemble physiological tissue architecture and reproduce interactions between cells and ECM. On the other hand, despite 3D cultures can be valid *in vitro* systems for studying chondrogenesis, our model needs further improvements and optimization for long-term cultures of chondrocytes after the production of well-organized and functional cartilage, in order to prevent dedifferentiating and/or hypertrophic drift events. Indeed, articular cartilage structure has a complex multi-layer architecture with different compositions and functions, and chondrocytes are clustered in small areas, known as lacunae. In our 3D system, we clearly observed randomly distributed depositions of acidic proteoglycans around cells, even if still not well organized as lacunae-like structures. However, *in vivo* reproduction of this peculiar architecture remains the main challenge in regenerative medicine of cartilage tissue.

## Conclusion

*In vitro* models to investigate cartilage regeneration processes are becoming increasingly important. Our results indicate that 3D high-density stem cell culture supplemented with 10 ng/ml of hTGF-β1 represents a simple and rapid 3D model of chondrogenic commitment. These 3D biomaterials are extremely versatile tools and could be used to evaluate the effects of several drugs and growth factors on cartilage regeneration and healing, as well as to explore the role of cytokines in leading chondrogenic commitment. Indeed, our 3D-culture systems highlighted a well-balanced expression of pro and anti-inflammatory cytokines suggesting their involvement in chondrogenic events. This trend was already observed for BM- but never described for WJ-3D systems and provided an important correlation between chondrogenic commitment that might be sustained by autocrine cytokine production. Our findings open interesting prospective for the use of hWJ-MSCs to develop 3D models for cartilage tissue engineering.

## Data Availability

The raw data supporting the conclusion of this article will be made available by the authors, without undue reservation.

## Abbreviations

hTGF-β1, human transforming growth factor β 1; hBM-MSCs, human Bone Marrow Mesenchymal Stromal Cells; hASC, human adipose stem cells; COL1A1, type I collagen; COL2A1, type II collagen; COL3A1, type III collagen; SOX9, SRY-Box transcription factor 9; ACAN, aggrecan; RT-qPCR, reverse transcription quantitative polymerase chain reaction; q-IF, semi-quantitative immunofluorescence.
